# Phytochemical Recovery from Purple Carrot Peels: Optimization and Characterization

**DOI:** 10.3390/plants15132036

**Published:** 2026-07-01

**Authors:** Alexandra Teodora Gheorghe (Mărtin), Oana Emilia Constantin, Elisabeta-Irina Geană, Doina Georgeta Andronoiu, Nicoleta Stănciuc, Iuliana Aprodu, Claudia Mureșan, Constantin Croitoru, Gabriela Râpeanu

**Affiliations:** 1 Department of Food Science, Engineering and Applied Biotechnology, Faculty of Food Science and Engineering, Dunarea de Jos University of Galati, 111 Domnească Street, 800201 Galati, Romania; teodora.martin83@gmail.com (A.T.G.); econstantin@ugal.ro (O.E.C.); georgeta.andronoiu@ugal.ro (D.G.A.); nstanciuc@ugal.ro (N.S.); iaprodu@ugal.ro (I.A.); constantin.croitoru@asas.ro (C.C.); 2 National R&D Institute for Cryogenics and Isotopic Technologies-ICSI Râmnicu Vâlcea, 4th Uzinei Street, 240050 Râmnicu Vâlcea, Romania; irina.geana@icsi.ro; 3 Faculty of Food Engineering, Tourism and Environmental Protection, Aurel Vlaicu University of Arad, 2 Elena, Dragoi Street, 310330 Arad, Romania; claudia.muresan@uav.ro; 4 Academy of Agricultural and Forestry Sciences, 61 Măraști Blvd, 011464 Bucharest, Romania

**Keywords:** purple carrot peels, polyphenols, antioxidant activity, solid–liquid extraction, response surface methodology, by-product valorization

## Abstract

Purple carrot peels are an abundant agro-industrial by-product rich in phytochemicals and have potential applications such as natural colorants and antioxidants. In the context of increasing interest in the valorization of vegetable processing residues, the recovery of bioactive compounds from such materials has become an important scientific and industrial objective. This study aimed to optimize the parameters of a solvent extraction process to maximize total anthocyanin content (TAC), total polyphenol content (TPC), and antioxidant activity (AA) from purple carrot peels. A Central Composite Design, combined with response surface methodology, was used to evaluate the effects of temperature, extraction time, ethanol concentration, and pH across 30 experimental runs. The optimal extraction conditions were identified as pH 4, an ethanol concentration of 50%, a temperature of 80 °C, and an extraction time of 35.88 min. Under the optimized extraction conditions, the experimental values obtained for TAC, TPC, and AA were 2.10 mg C3G/g D.W., 20.60 mg GAE/g D.W, and 19.02 mMol Trolox/g D.W, respectively, which were in close agreement with the predicted values of 2.10 mg C3G/g D.W., 20.30 mg GAE/g D.W, and 19.05 mMol Trolox/g D.W. The good agreement between predicted and experimental values confirmed the adequacy and predictive capability of the developed response surface models. A total of 32 phenolic compounds were characterized by UHPLC–MS/MS, with shikimic acid identified as the dominant compound, highlighting the chemical diversity and abundance of bioactive phenolics in purple carrot peels. The results demonstrate that optimization of extraction parameters significantly enhances the efficiency of conventional solid–liquid extraction, enabling the effective recovery of antioxidant compounds. The proposed approach supports the sustainable valorization of purple carrot by-products as cost-effective sources of natural pigments for food industry applications.

## 1. Introduction

In vegetable processing, carrot peels are by-products that allow valorizing some of the biologically active compounds in them with biological functions for the human body [1,2,3]. Even though they have thin skin, carrots contain significant amounts of the most essential nutrients. Anthocyanins, mainly present in purple roots, and flavonoids, found in high quantities in black carrots, have been extensively investigated and reviewed in the literature [4,5,6]. Purple carrots (*Daucus carota* ssp. sativus var. atrorubens Alef.) cultivar Deep Purple are characterized by their dark purple roots due to high concentrations of anthocyanins. Interest in purple carrot anthocyanins has been increasing in recent years. They are considered valuable natural food colorants essential to human health, particularly for their antioxidant activity [7].

Recently, research has focused on identifying varieties of multicolored carrots, which are rich in phytochemicals, as consumers become increasingly aware of the need to adopt a healthy diet. The purple carrot is being valorized as a food colorant due to its high storage and thermal processing stability [8].

In addition to anthocyanins, polyphenols are compounds that play a key role in mitigating oxidative stress and inflammatory processes in the body [9]. The pigments in black carrots make them healthier than yellow, orange, or red carrots [10]. To overcome the challenges in conventional extraction methods, innovative extraction techniques such as microwave-assisted extraction, ultrasound-assisted extraction, pulsed electric field, and surface extraction techniques are being investigated [11]. However, these techniques require expensive equipment or high energy consumption, with conventional solvent extraction remaining the easiest and the most sustainable choice. In this context, intense studies have been conducted to elucidate the structural and functional mechanisms for separating pigments from plant residues [12]. However, the optimization of the anthocyanin extraction method relies on appropriate mathematical models to justify the extraction yield and high quality [13].

Generally, the choice of extraction parameters, such as temperature, solvent concentration, solvent–liquid ratio, pH, extraction time, or particle size of the plant matrix, is essential to optimize the recovery of bioactive compounds [14]. Choosing the appropriate solvent may enhance the extraction rate, time, effectiveness, and solvent consumption. The extraction of bioactive compounds from plant matrix is a multi-step process that has been the subject of much research. The extraction parameters, such as solvent concentration, pH, extraction duration, and temperature, play a significant role in this process. The efficiency of the extraction process depends on the nature of the polyphenolic compounds present in the plant powders, as well as the choice of a suitable solvent that can reduce the extraction consumption and extraction time [15]. The solubility of polyphenols can be explained by their stereochemistry (polar and non-polar fragmentation of the molecules) and the intermolecular forces (hydrogen bonds) formed between them and the solvent [16,17]. The ethanol solution concentration influences the extraction yield of phenolic compounds and the degradation rate of bioactive compounds.

Purple carrot peel was selected as the extraction matrix because it is an underutilized agri-food by-product with potential value as a source of anthocyanins, phenolic compounds, and natural antioxidants. Unlike conventional orange carrots, which are mainly recognized for their carotenoid content, purple carrots are characterized by the presence of anthocyanin pigments and other phenolic compounds. Therefore, optimizing the extraction conditions for this specific by-product is of practical relevance to its valorization, while the UHPLC–MS/MS analysis is interpreted as providing supportive qualitative profiling.

The objective of the current experimental study was to optimize the extraction conditions of polyphenolic compounds with high antioxidant activity from purple carrot peel powder using the conventional solvent method. Using a Central Composite Design (CCD) projection matrix with 30 experimental values, the investigation varied the extraction parameters—time, temperature, ethanol concentration, and pH—to obtain extracts rich in biologically active compounds with strong antioxidant activity. Although several assisted extraction techniques have been applied to purple carrot matrices, conventional solid–liquid extraction remains relevant due to its simplicity, low equipment requirements, scalability, and compatibility with food-grade solvents. Therefore, the present study aimed to optimize this accessible extraction approach to valorize purple carrot peel, with particular emphasis on the simultaneous recovery of anthocyanins, polyphenols, and antioxidant compounds.

## 2. Results and Discussion

### 2.1. Fitting the Response Surface Models

Bioactive compounds are typically extracted under the influence of a range of variables and may have either synergistic or antagonistic effects on one another. Consequently, to obtain the highest-quality, most efficacious extract, it is imperative to investigate the optimal values of several factors and determine the most favorable conditions. In this experimental study, the extraction parameters for phytochemicals from purple carrot peels were optimized using a Central Composite Design (CCD) projection matrix and the response surface method. The responses were anthocyanin, polyphenols, and antioxidant activity in purple carrot by-products.

The optimized extraction conditions obtained in this study should therefore be interpreted as a practical, technically accessible alternative for recovering bioactive compounds from purple carrot peel, especially in contexts where advanced extraction technologies are not available or economically justified.

Table 1 presents the complete CCD matrix for extraction optimization using the variables time, temperature, pH, and ethanol concentration.

The second-order polynomial model was used to fit the experimental data, and the resulting equation was evaluated to determine if it could accurately characterize the variability in the responses. This was accomplished by evaluating the coefficients of multiple determination and conducting variance analyses. Table 2 displays the regression coefficients for the intercept, linear, quadratic, and cross-product terms.

Table 3 presents the ANOVA results for the reduced quadratic response surface model, together with the coefficients of multiple determination (R^2^) for TAC, TPC, and AA.

### 2.2. Influence of the Extraction Parameters on TAC

This experimental study aimed to optimize TAC extraction conditions from purple carrot peel powders by identifying the optimal extraction parameters. Thus, the TAC ranged from 0.37 to 4.33 mg C3G/g D.W. in the experimental model. Similar results for TAC in 12 carrot varieties were obtained in a study by Ma et al. [18] that ranged from 0.03 mg/g D.W. to 6.18 mg/g D.W. Moreover, Leja et al. [19] obtained a high amount of anthocyanins for the purple carrot roots, with an average of 64.9 mg 100/g fresh weight (F.W). The authors also demonstrate that the contents of phenylpropanoids and flavonols are 14- and 8-fold higher, respectively, than in other colored roots.

In Table 3, in the case of the TAC, a model F-value of 49.81 implies that the model is significant. *p*-values less than 0.05 also indicate that the model terms are significant: A, C, D, AB, AC, AD, BC, BD, CD, A^2^, C^2^, D^2^. The predicted coefficient of determination, R^2^pred = 0.9092, is consistent with the adjusted coefficient of determination, adjusted R^2^adj = 0.9563. The extraction of TAC from carrot peels was positively influenced by the interactions between temperature and pH (AD) and ethanol and pH (CD), as indicated in Table 3.

**Figure 1:**
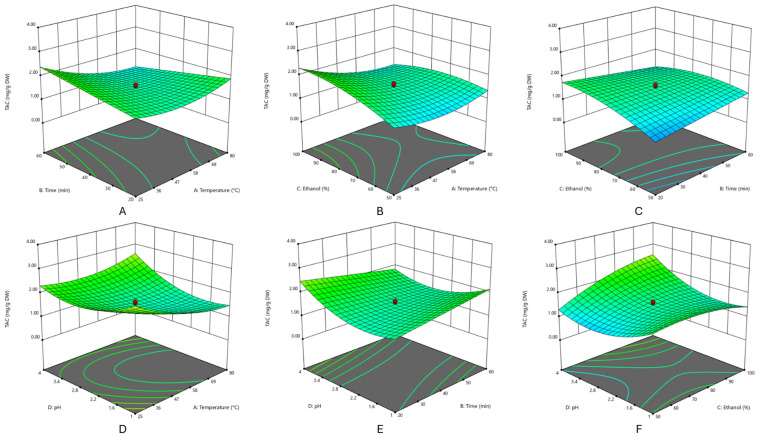
Response surface area plots of TAC as a function of extraction time and temperature (**A**); ethanol concentration and temperature (**B**); ethanol concentration and extraction time (**C**); pH and temperature (**D**); pH and extraction time (**E**); pH and ethanol concentration (**F**).

Figure 1 shows the 3D response surface plots representing the interactive effects of the independent variables on TAC content. Among the tested factors, extraction temperature, solution pH, and ethanol concentration had a greater impact on TAC, whereas extraction time showed a comparatively lower influence. The plots (A, B) indicate that TAC increases with higher ethanol concentrations and, at lower extraction temperatures, in proportion to extraction time. The increase in temperature, concomitant with ethanol concentration, negatively influences the extraction results (regression coefficient b_13_ = −0.2739; Table 2). Our results agree with those of Khazaei et al. [20], who found increases in TAC with increases in ethanol concentration and extraction time. Cacace et al. [21] conducted a similar investigation examining anthocyanins extracted from blackcurrant and the relationship between ethanol concentration and temperature over a 150-min extraction period. They noted that increasing the ethanol content (from 20 to 85%) raised the critical temperature of anthocyanins from 25 to 35 °C. It was discovered that the extraction efficiency improved at all temperatures and then declined as the ethanol percentage increased to 60% In contrast, Backes et al. [22] showed that ethanol in high concentrations in combination with medium temperatures favors optimal anthocyanin extraction (100% ethanol acidified with citric acid, mixed and centrifuged for about 14 min at 36 °C was the optimal analytical factor for growth). According to several previous studies, higher temperatures cause anthocyanin degradation [20,23].

Alternatively, the surface plot (C) reveals that an inversely proportional increase in ethanol concentration over time is necessary for reliable results. Thus, as the solvent concentration increases, the extraction time must be decreased. The maximum anthocyanin content could be reached at pH = 1, an ethanol concentration of almost 50%, and an extraction time of about 60 min (surface plots D, F). Surface plot (E) indicates that suitable extraction results are obtained by preserving the pH and the temperature constant. It can also be seen that optimal extraction values can be obtained by increasing the temperature while maintaining a low pH. Comparable results were obtained for purple carrot ‘Pupur’ and ‘Mayami shokoladnaya’ [24]. The data indicated that a rise in pH results in a decrease in extraction efficiency, obtaining for 0.1 M water solution of HCl (pH = 1) a TAC of 0.111 g and 0.232 g/100 g F.W. for cultivars I and II, respectively, compared with 0.01 M water solution of HCl (pH = 2), in which the TAC content was 0.147 and 0.152 g/100 g F.W. [24].

### 2.3. Influence of the Extraction Parameters on TPC

This experimental study aimed to identify the optimal extraction model to maximize the extraction of phytochemical compounds, such as polyphenols, from purple carrot peel. Thus, the TPC content ranged from 7.22 to 21.67 mg GAE/g D.W. based on the experimental model. Comparable results were observed for total phenolic content across 12 carrot varieties, with values ranging from 7.25 to 33.25 mg/g D.W. [18]. The mean TPC values for root phenotypes across the 29 examined carrot accessions over two years of harvest ranged from 0.172 to 4.669 mg GAE/g F.W. in 2018 and from 0.215 to 4.310 mg GAE/g F.W. in 2019 [25].

Table 3 shows that, for TPC, the model F-value of 76.51 indicates that the model is significant. *p*-values below 0.05 indicate that the model terms are statistically significant. In this case, A, D, AC, AD, BC, BD, CD, B^2^, C^2^, and D^2^ are the significant terms of the model. Among them, ethanol negatively influences the extraction temperature and the temperature concomitant with ethanol concentration (regression coefficient b_13_ = −1.38; Table 2). The predicted coefficient of determination, R^2^pred = 0.9312, is consistent with the adjusted coefficient of determination, R^2^adj = 0.9690.

**Figure 2:**
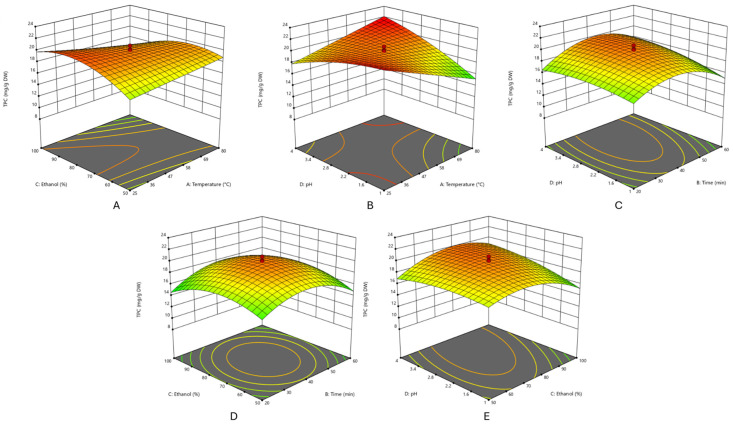
Response surface area plots of TPC as a function of ethanol concentration and temperature (**A**); pH and temperature (**B**); pH and extraction time (**C**); ethanol concentration and time (**D**); pH and ethanol concentration (**E**).

Figure 2 shows three-dimensional plotted response surfaces and contour plots depicting the correlative effect of selected factors on determining TPC content. From surface plots A and D, it can be observed that these compounds are optimally extracted in the presence of a high concentration of ethanol (60–100%) at a maximum temperature of 60 °C, and an extraction time of 40 min. In a study conducted by Cacace et al. [26] regarding the optimization of phenolic compound extraction from milled berries, it was revealed that the maximum yield of phenolic compounds was achieved using 60% ethanol for 150 min, while changing in ethanol concentration resulted in diminished extraction rates; however, beyond this concentration, decreased efficiency was observed, a finding corroborated by our work. Moreover, Mantiniotou et al. [27] established that the most effective conditions for polyphenol recovery from carrot peels (*Daucus carota* L.) were achieved through conventional extraction, which involved 25% *v*/*v* ethanol, high temperatures, and extended extraction time (80 °C for 150 min). The response surface area plots (B, C) show the extraction related to pH and temperature and indicate that the temperature 40–52 °C positively influences the extraction, provided that a low pH (pH-2.5) is maintained. Surface plots D and E show that TPC is extracted in large amounts at a pH between 2.5 and 4, an ethanol concentration of 70–80%, and an extraction time of 30–40 min. Moreover, according to Sabahi et al. [28] the extraction time is crucial in the context of polyphenolics, as increasing the interaction duration of the solvent (70% ethanol) with solid materials may improve the dispersion of these substances.

### 2.4. Influence of the Extraction Parameters on AA

In the extraction optimization study to determine AA, the aim was to identify an optimal model for correlating the extraction parameters of purple carrot peels. As shown in Table 1, the concentration of AA ranged from 7.20 to 22.79 mMol Trolox/g/g D.W. The results are lower than those obtained by Yildiz et al. [29] on black carrot samples from the Hatay region. The AA obtained for the Hatay variety was 15.13 ± 0.22 µmole Trolox/g, and for the Konya variety, 20.98 ± 0.33 µmole Trolox/g. In another study conducted by Kosewski et al. [30], the carrot sample revealed an AA of 0.42 μM Trolox/g.

Based on the variables in the extraction medium, the regression equations developed following ANOVA explained the AA values of the extract obtained from purple carrot peels. The F value of the model, 91.58, indicates that the model is significant, with A, B, C, D, AB, AC, AD, CD, B^2^, C^2^, and D^2^ being the considerable model terms. According to the regression model for the DPPH free radical scavenging activity, the coefficient of determination (R^2^) was 0.98. This indicates that the current model can specify only 0.02 of the variance in AA. The predicted coefficient of determination R^2^pred of 0.9412 is consistent with the adjusted coefficient of determination R^2^adj of 0.9717 (Table 3). The regression coefficients in Table 2 indicate that both temperature and pH have positive effects on AA. The extraction time and ethanol concentration had a detrimental effect on AA. The relationship between temperature and ethanol concentration (AC) notably diminished the AA of carrot peel powder. The interaction between temperature and time (AB) showed a slight positive effect. The interactions involving the quadratic term of ethanol concentration (C^2^) had a significant positive impact on AA.

**Figure 3:**
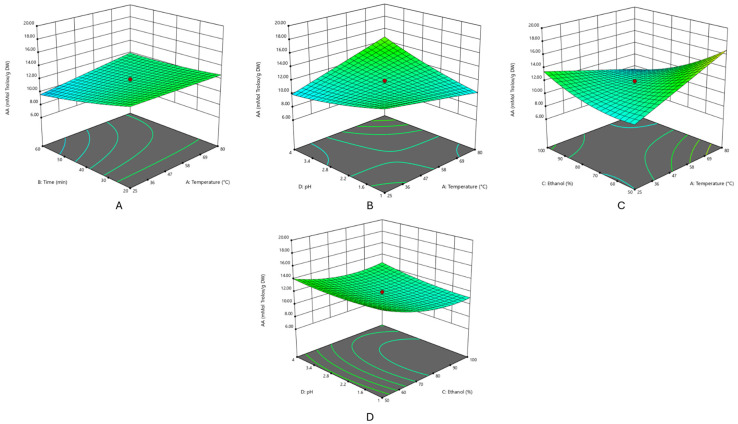
Response surface area plots of AA versus time and temperature (**A**); pH and temperature (**B**); ethanol concentration and temperature (**C**); pH and ethanol concentration (**D**).

Figure 3 shows response surfaces depicting the correlational effect of selected factors on AA. It can be seen from plots (A) and (B) that pigments such as polyphenolic compounds exhibit the highest AA under high-temperature conditions of 80 °C and pH = 4, with time not significantly influencing the values. The extraction is positively influenced by solvent concentration (50%) and extraction temperature (80 °C), as shown in surface plots C and D.

Our data correlate with those of Stübler et al. [31], who investigated the stability of polyphenols in strawberry puree by various thermal processing techniques, with significant increases in the AA of strawberries in a water mixture being directly proportional to temperature increase. Therefore, they concluded that heat treatment can provide the energy required to disrupt interactions within the polyphenol matrix, thereby enhancing AA.

**Figure 4:**
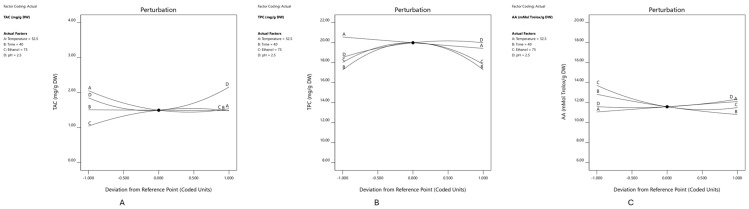
Perturbation plots describing the effect of each independent variable on the extraction of TAC (**A**), TPC (**B**), and AA (**C**).

Analysis of deviations from the baseline reveals that a high or curved slope for a given factor reflects sensitivity of the response to that factor. A relatively flat line indicates a lack of sensitivity to variations in that factor. The main factor affecting TAC extraction is the solution pH (Figure 4A, curve D), followed by temperature (Figure 4A, curve A) and ethanol concentration (Figure 4A, curve C).

According to the perturbation plot describing each independent variable’s effect, temperature strongly influenced TPC extraction (Figure 4B, curve A). In contrast, AA is influenced by ethanol concentration and extraction time (Figure 4C, curves C and B).

### 2.5. Optimization and Validation of the Extraction Parameters

**Figure 5:**
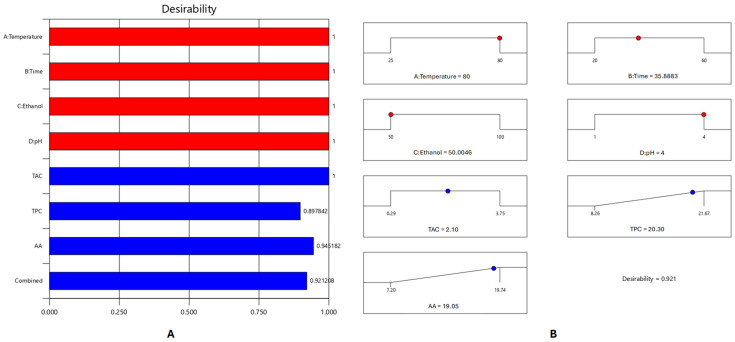
Desirability plot (**A**) and ramps plot (**B**) of process parameters and responses.

To confirm the model equation, the model suggested the best factors to maximize the response desirability (Table 4). A desirability value close to 1 (0.921) indicated that all selected conditions were in a suitable combination (Figure 5).

Solvent extraction was conducted under the optimal conditions specified by the software to evaluate their compatibility with laboratory conditions (Table 4).

Response surface methodology with a central composite design was successfully used to optimize the extraction conditions, obtaining the following parameters: pH 4, ethanol concentration 50%, temperature 80 °C, and extraction time 35.88 min. The model estimated the maximum TAC of 2.10 mg C3G/g D.W., TPC of 20.30 mg GAE/g D.W., and AA of 19.05 mMol Trolox/g D.W., respectively (Table 4). The total experimental values obtained under laboratory conditions are closely aligned with the model’s predicted value, indicating a strong fit to the predicted data. These findings are in accordance with Park et al. [32] that had a TPC calculated to be a maximum of 18.1 mg GAE/g DM under the optimal TPC extraction conditions for purple carrots, which were anticipated to be 32.5 min, 76.3 °C, and 38.0% ethanol based on RSM.

### 2.6. Content by UHPLC-MS/MS Analysis for Phenolic Compounds

Polyphenolic compounds are secondary metabolites of plants, essential for the color, nutritional value, antioxidant activity, and sensory attributes of foods [33]. Compared to other pigmented carrot varieties, purple carrot cultivars contain a higher concentration of phenolic compounds [34]. Purple carrot peel is the extraction matrix due to its phytochemicals and status as an underutilized by-product. Unlike orange carrots rich in carotenoids, purple carrots contain anthocyanins and phenolics. Optimizing extraction conditions, considering solvent, pH, temperature, and time, is vital for maximizing the recovery and stability of these antioxidants.

**Figure 6:**
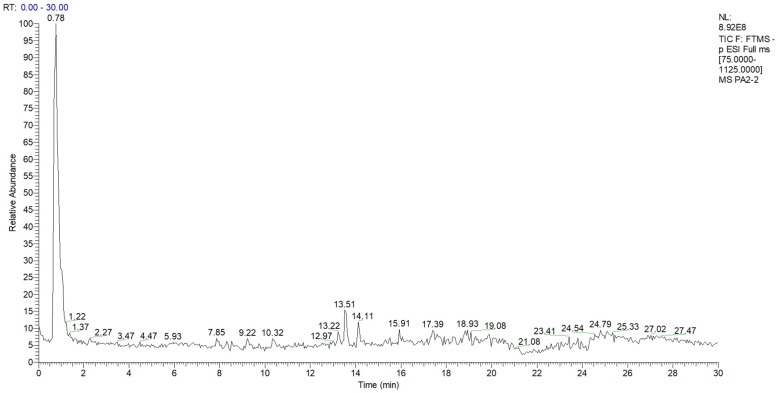
UHPLC–MS/MS total ion current (TIC) chromatogram of purple carrot peel powder recorded in negative electrospray ionization mode.

The UHPLC–MS/MS analysis provided qualitative support for the phytochemical profile of the optimized extract. The polyphenolic compositions were analyzed using UHPLC-MS/MS, and the results are presented in Table 5 and Figure 6.

Forty-four phenolic compounds (15 phenolic acids and phenolic acid derivatives, one tannin, 21 flavonoids, 3 stilbenoids, four acylated anthocyanins, and two dihydrochalcones) were detected from purple carrot peel powder. The dominant compound was shikimic acid (87.40 ± 8.04 mg/kg d.w.), indicating its major contribution to the phytochemical composition. Among phenolic acids, 3,4-dihydroxybenzoic acid and 4-hydroxybenzoic acid were quantified at relevant levels, while most hydroxycinnamic acids were present only in low amounts or below the quantification limit. The discovered phenolic acids in purple carrot types exhibited similarities to those documented in the literature [19,35,36]. The study conducted by El-Moslemany et al. [37] identified numerous polyphenolic compounds from purple carrot extract (*Daucus carota*), including catechin, ferulic acid, vanillic acid, sinapic acid, rutin, cinnamic acid, apigenin, and kaempferol. The results obtained were in agreement with those reported by [35,38,39] that carrots are rich in phenolic acids, mainly hydroxycinnamic acids and their derivatives, such as p-hydroxybenzoic, caffeic, and chlorogenic, as well as in anthocyanins, a class of flavonoids.

Important flavonoids included quercetin, isorhamnetin, naringin, rutin, catechin, and epigallocatechin gallate, although generally at moderate or low concentrations. Ellagic acid was also notable, suggesting the presence of tannin-derived phenolics. Anthocyanins were represented by oenin, detected at a low level, while stilbenoids such as cis-polydatin and isorhapontigenin were also quantified. Several acylated anthocyanins, mainly cyanidin- and peonidin-based derivatives, were also detected. These compounds are particularly relevant because they are characteristic pigments of purple carrot and contribute to its color stability and functional properties [40].

## 3. Materials and Methods

### 3.1. Purple Carrot Peel Powder Preparation

Purple carrots (*Daucus carota* ssp. sativus var. atrorubens Alef. Deep Purple cultivar) cultivated in Spain were provided from a hypermarket in Galati County. In order to obtain the peel powder, the following steps were succeeded: washing the purple carrot roots under cold water to remove impurities from their surface, rinsing the roots with ultrapure water, followed by wiping them repeatedly with paper towels, removing the peel in a thin layer, avoiding cutting the pulp, followed by drying the peels by freeze-drying for 48 h, at −42 °C and pressure 0.10 mBar, using freeze-dryer (CHRIST Alpha 1-4 LD plus, Martin Christ Gefriertrocknungsanlagen GmbH, Osterode am Harz, Germany). Subsequently, the lyophilized peels were finely ground for 1 min using a razor-blade grinder. Thus, the powder collected was stored in glass containers in a dark place at room temperature.

### 3.2. Chemicals

Ethanol, glacial acetic acid, sodium acetate, hydrochloric acid, NaOH, Folin–Ciocalteu reagent, Na_2_CO_3_, KCl, NaNO_2_, AlCl_3_, DPPH, TROLOX, and gallic acid were obtained from Sigma Aldrich Steinheim (Darmstadt, Germany). All other reagents used in the experiments were of analytical grade.

### 3.3. Conventional Solid/Liquid Extraction

For a conventional extraction, 1 g of purple carrot peel powder and 9 mL of ethanol were used in different concentrations of 0–100%, and each extraction was acidified with glacial acetic acid. The purple carrot powder-to-solvent ratio was 1:10. pH correction with NaOH or HCl was performed before extractions. Extractions were achieved at different temperatures between 25 °C and 107.5 °C, with different extraction times (0–80 min); the samples were previously homogenized on an orbital shaker (SI-300R Medline Scientific, Chalgrove, UK) at 250 rpm for 15 min. Centrifugation of the mixture for 10 min at 6000 rpm at 4 °C with a Hettich Universal 320R centrifuge (Tuttlingen, Germany) was followed, after which the supernatant was phytochemically characterized. For extraction runs performed at elevated temperature, including the axial CCD conditions, samples were processed in closed extraction vessels to minimize solvent evaporation. The temperature was controlled using the heating equipment and monitored during extraction. After the extraction time elapsed, the vessels were cooled before opening to reduce solvent loss and limit changes in solvent composition.

### 3.4. Extract Characterization

- Total anthocyanin content (TAC)

TAC was determined using the differential pH method with two reagents: a buffer solution of potassium chloride (pH = 1.0) of concentration 0.025 M and a buffer solution of sodium acetate (pH = 4.5) of concentration 0.4 M. Absorbances were subsequently measured at 520 and 700 nm using a UV–VIS spectrophotometer (Libra S22, Biochrom, Cambridge, UK). The TAC was expressed as mg cyanidin 3-O-glucoside (C3G)/g D.W. and was calculated according to Equation (1) as described by Lee et al. [41], with minor modifications:

(1) $$\mathrm{TAC}\;\mathrm{mg}/g\;=\;\frac{A\times MW\times DF\times Vt}{\varepsilon \times l\times m}$$

where: A is the difference between the absorbances read for the two reagents (A_520nm_–A_700nm_) at pH 1.0—(A_520nm_–A_700nm_) at pH 4.5; MW—molecular weight (449.2 g/mol) of cyanidin-3-glucoside; DF—dilution factor; Vt—total volume (mL); ε—molar extinction coefficient (26.900) of cyanidin-3-glucoside; l—cuvette width, 1 cm; m—mass of the sample taken for analysis (g).

- Total phenolic content (TPC)

The TPC content was determined by the Folin–Ciocalteu colorimetric technique described by [42]. This technique is based on the chemical reduction of a mixture of wolfram and molybdenum oxides called the Folin–Ciocalteu reagent. The resulting compounds have an absorbance maximum of 765 nm. The concentration of polyphenolic compounds was quantified as mg gallic acid equivalents (GAE)/g dry weight (D.W.), utilizing the equation derived from the gallic acid calibration standard curve (y = 1.6991x − 0.0256, R^2^ = 0.9837).

- Antioxidant activity (AA)

The antioxidant activity was determined by the DPPH free radical scavenging method, as described by [42]: 100 µL of extract was mixed with 3.9 mL of DPPH solution and kept in the dark at room temperature for 90 min. Subsequently, absorbance was read at 515 nm, and the results were quantified using a Trolox calibration curve. It was expressed in mMol Trolox Equivalents (TE)/g D.W. The Trolox concentration for the standard curve ranged from 10 to 100 ppm, yielding the equation y = 0.45x + 0.0075 with R^2^ = 0.993.

- Determination of Phenolic Compound Content by UHPLC-MS/MS

For the determination of phenolic profile by UHPLC-MS/MS, the purple carrot powder was extracted by a conventional solid/liquid extraction method using a closed system (Mars VI, CEM Corp., Matthews, NC, USA) equipped with 100 mL closed vessel reactions (GreenChem) and integrated sensors for temperature and pressure, as well as steering and cooling systems. The extraction conditions were as follows: extraction solvent: 50% ethanol, extraction time: 36 min, temperature: 80 °C. At the end of the extraction program, the vessels were allowed to cool for 30 min at room temperature. Then, the extract was centrifuged for 15 min at 4 °C and 10,000 rpm (Hettich Rotina 380 R Centrifuge, Tuttingen, Germany) to separate the hydroethanolic extract from the solid residue. For analytical determinations, an aliquot of the sample was evaporated to dryness in a stream of nitrogen using a TurboVap LV (Biotage, Uppsala, Sweden), reconstituted with a 1 mL mixture of water:methanol (80:20), filtered through a 0.45 µm hydrophilic membrane filter, and subjected to instrumental analysis. The identification and quantification of the phenolic compounds in the extract were performed with a system consisting of an ultra-high-performance liquid chromatograph UltiMate 3000 UHPLC (ThermoFisher Scientific, Bremen, Germany) coupled to a Q Exactive Focus mass spectrometer Focus Hybrid Quadrupole—OrbiTrap (Thermo Fisher Scientific) equipped with HESI. Chromatographic separation of phenolic compounds was performed using a Kinetex C18 column (100 × 2.1 mm; 1.7 μm particle diameter) maintained at 30 °C, under a gradient elution of two mobile phases, A (0.1% formic acid in water) and B (0.1% formic acid in methanol), at flow rates of 0.3 and 0.4 mL/min, as previously optimized [43]. The mass spectrometer was operated in negative mode, in a range between 100–1000 *m*/*z*, at a resolution of 70,000, under the previously optimized HESI-source parameters (spray voltage 2.8 kV, capillary temperature 320 °C, auxiliary gas heater temperature 413 °C, sheath and auxiliary gas flow (N_2_) 35 and 10 arbitrary units) [43]. Identification and quantification of the phenolic compounds in the extract were performed based on their spectral characteristics, including mass spectra, accurate mass measurements, and characteristic retention times relative to external standard solutions. Fragmentation studies by a data-dependent scan with collision-induced dissociation (CID) (normalized collision energy of the CID cell set at 25, 35, and 45 eV) were performed to confirm the identified compounds. Instrument calibration was performed over the concentration range of 0–2000 μg/L for each phenolic compound using serial dilutions in methanol from the 10 mg/L calibration standard mixture. All stock and working solutions were stored in the dark at 4 °C. Xcalibur software (Version 4.1) was used for instrument control, data acquisition, and data analysis.

### 3.5. Experimental Design

A modified Central Composite Design (CCD) was used to optimize the extraction of biologically active compounds and the determination of AA using Design Expert software (version 12, Stat-Ease Inc., Minneapolis, MN, USA). Four independent variables were investigated: extraction temperature (A, °C), extraction time (B, min), ethanol concentration (C), and pH (D). The experimental ranges were selected based on preliminary conventional extraction trials and literature data, considering the practical feasibility of the extraction process, solvent stability, and the sensitivity of anthocyanins and phenolic compounds to pH and temperature.

The CCD consisted of 16 factorial points, 8 axial points, and 6 center-point replications, resulting in 30 experimental runs. Table 6 shows the maximum and minimum values of the independent variables analyzed in the experimental design, both in their current and coded forms. In addition, the CCD generates a quadratic regression model for the response variables, thus contributing to process optimization. Because some experimental conditions were limited by practical and chemical feasibility, a constrained modified CCD was applied instead of a fully symmetrical rotatable CCD. Therefore, the upper axial level for the ethanol factor was constrained at the feasible maximum value.

The response variables were fitted using the following second-order polynomial model (2):

(2) $$R\;=\;b_{0}+\sum_{i}^{n}b_{i}\cdot x_{i}+\sum_{i=1}^{n}b_{ii}{\cdot x}_{i}^{2}+\sum b_{ij}\cdot x_{i}\cdot x_{j},$$

R—predicted response, b_0_—represents the constant or free term of the model, b_i_, b_ii_, and b_ij_—the regression coefficients of the linear, quadratic, and interaction terms, x_i_ and x_j_—independent variables analyzed, and n—number of factors.

The validation experiment was performed under the global optimum conditions predicted by the multi-response desirability function, rather than under the individual optimum conditions for TAC, TPC, or AA separately. This strategy was selected because the study aimed to identify a single practical extraction protocol that simultaneously achieves high recovery of anthocyanins and polyphenols and strong antioxidant activity.

The statistical adequacy of the fitted second-order polynomial models was evaluated using analysis of variance, coefficient of determination, adjusted and predicted R^2^ values, pure error, and lack-of-fit analysis.

## 4. Conclusions

This study demonstrates that purple carrot peel is a valuable source of natural pigments and antioxidant compounds. Rather than introducing a new extraction technology, the study contributes by establishing a matrix-specific conventional extraction model that integrates the effects of pH, ethanol concentration, temperature, and time on anthocyanin recovery, phenolic extraction, and antioxidant activity. Using a Central Composite Design coupled with response surface methodology enabled the identification of optimal extraction parameters that significantly enhanced the recovery of anthocyanins and polyphenols, achieving notable levels of TAC (2.10 mg C3G/g D.W.), TPC (20.30 mg GAE/g D.W.), and AA (19.05 mMol Trolox/g D.W.).

The optimized conventional solid–liquid extraction method effectively recovers bioactive compounds from purple carrot peels. These results indicate that well-tuned conventional techniques could serve as practical and accessible means to valorize carrot processing by-products. Such findings highlight the sustainable potential of utilizing these by-products as cost-efficient sources of natural food colorants and functional ingredients for the food industry. However, further comparative research is necessary to fully evaluate the approach’s advantages.

## Figures and Tables

**Table 1:** Complete CCD Matrix for extraction optimization.

Run	A	B	C	D	TAC, mg C3G/g D.W.	TPC, mg GAE/g D.W.	AA, mMol Trolox/g D.W.
1	80	20	100	4	3.75	14.94	12.71
2	52.5	40	75	2.5	1.64	20.16	12.01
3	80	60	50	4	1.61	17.53	19.23
4	52.5	40	75	2.5	1.31	20.81	11.44
5	25	60	100	1	2.96	16.23	12.84
6	25	20	50	4	1.00	9.93	10.35
7	80	60	50	1	1.76	11.47	15.62
8	25	60	100	4	2.92	15.94	11.72
9	107.5	40	75	2.5	0.37	18.00	12.81
10	25	20	50	1	1.45	17.28	15.16
11	80	20	50	1	1.41	14.57	15.41
12	52.5	40	75	2.5	1.60	19.33	11.31
13	52.5	40	75	2.5	1.59	19.96	11.43
14	25	60	50	4	1.48	10.44	8.00
15	52.5	40	75	0	2.97	16.20	11.59
16	25	60	50	1	3.35	16.24	11.95
17	80	20	50	4	2.32	18.23	19.74
18	80	60	100	1	0.49	8.26	7.20
19	52.5	40	100	2.5	0.89	11.12	13.76
20	25	20	100	4	3.20	14.78	14.33
21	25	20	100	1	2.39	17.93	17.03
22	80	20	100	1	0.94	8.64	8.37
23	52.5	40	75	2.5	0.29	12.30	17.59
24	52.5	40	75	2.5	3.70	18.90	14.15
25	52.5	0	75	2.5	1.29	8.73	15.01
26	52.5	80	75	2.5	1.24	9.55	10.01
27	80	40	75	2.5	2.97	21.67	11.23
28	52.5	40	75	2.5	1.63	20.03	11.47
29	80	60	100	4	1.73	18.86	12.25
30	52.5	40	75	2.5	1.66	20.06	11.05

A: extraction temperature (°C); B: pH; C: ethanol concentration (%); D: extraction time (min); TAC: total anthocyanin content (mg C3G/g D.W.); TPC: total phenolic content (mg GAE/g D.W.); AA: antioxidant activity (mMol Trolox/g D.W).

**Table 2:** Regression coefficients of the reduced quadratic model for the responses TAC, TPC, and AA from purple carrot peel powder.

Factor	Regression Coefficient	Coefficient Estimate		
		TAC	TPC	AA
Intercept	b_0_	1.49	19.98	11.58
A-Temperature	b_1_	−0.2578	−0.5694	0.5197
B-Time	b_2_	−0.0112	0.0133	−1.01
C-Ethanol	b_3_	0.2175	−0.1025	−1.11
D-pH	b_4_	0.1534	0.7307	0.3754
AB	b_12_	−0.3438	-	0.6521
AC	b_13_	−0.2739	−1.38	−2.50
AD	b_14_	0.3984	2.70	1.87
BC	b_23_	−0.2621	0.4578	-
BD	b_24_	−0.3061	0.6948	-
CD	b_34_	0.3998	1.06	0.3990
A^2^	b_11_	0.3017	-	-
B^2^	b_22_	-	−2.71	0.2393
C^2^	b_33_	−0.2365	−2.07	1.03
D^2^	b_44_	0.5093	−0.7342	0.3894

A: extraction temperature (°C); B: pH; C: ethanol concentration (%); D: extraction time (min); TAC: total anthocyanin content (mg C3G/g D.W.); TPC: total phenolic content (mg GAE/g D.W.); AA: antioxidant activity (mMol Trolox/g D.W).

**Table 3:** ANOVA for the reduced quadratic model of TAC, TPC, and AA.

Source	TAC				TPC				AA			
	SS	df	MS	F/*p*	SS	df	MS	F/*p*	SS	df	MS	F/*p*
Model	24.58	13	1.89	49.81 **	490.70	12	40.89	76.51 **	261.13	11	23.74	91.58 **
A	1.57	1	1.57	41.35 **	7.67	1	7.67	14.34 *	6.39	1	6.39	24.64 **
B	0.0030	1	0.0030	0.0792 ***	0.0042	1	0.0042	0.0079 ***	24.58	1	24.58	94.82 **
C	1.14	1	1.14	29.91 **	0.2524	1	0.2524	0.4722 ***	29.60	1	29.60	114.20 **
D	0.5259	1	0.5259	13.86 **	11.93	1	11.93	22.32 *	3.15	1	3.15	12.15 *
AB	1.89	1	1.89	49.81 **	-	-	-	-	6.80	1	6.80	26.25 **
AC	1.20	1	1.20	31.62 **	30.52	1	30.52	57.09 **	99.65	1	99.65	384.45 **
AD	2.54	1	2.54	66.90 **	116.72	1	116.72	218.38 **	56.00	1	56.00	216.06 **
BC	1.10	1	1.10	28.97 **	3.35	1	3.35	6.27 *	-	-	-	-
BD	1.50	1	1.50	39.51 **	7.72	1	7.72	14.45 *	-	-	-	-
CD	2.56	1	2.56	67.37 **	17.85	1	17.85	33.40 **	2.55	1	2.55	9.83 *
A^2^	2.38	1	2.38	62.59 **	-	-	-	-	-	-	-	-
B^2^	-	-	-	-	207.62	1	207.62	388.45 **	1.61	1	1.61	6.22 *
C^2^	1.58	1	1.58	41.59 **	120.96	1	120.96	226.31 **	29.87	1	29.87	115.24 **
D^2^	5.62	1	5.62	147.98 **	11.65	1	11.65	21.80 **	3.28	1	3.28	12.65 *
Residual	0.6073	16	0.0380		9.09	17	0.5345		4.67	18	0.2592	
Lack of Fit	0.5218	11	0.0474	2.78 ***	7.97	12	0.6639	2.96 ***	4.17	13	0.3209	3.24 ***
Pure Error	0.0855	5	0.0171		1.12	5	0.2240		0.4945	5	0.0989	
Cor Total	25.18	29			499.79	29			265.79	29		
R^2^	0.9759				0.9818				0.9824			
R^2^adj	0.9563				0.9690				0.9717			
R^2^pred	0.9092				0.9312				0.9412			

SS—Sum of Squares; df—degrees of freedom; MS—Mean Square; F—F-value; *p*—*p*-value; R^2^adj—Adjusted R^2^; R^2^pred—Predicted R^2^; * Significant at *p* < 0.01, ** Significant at *p* < 0.001, *** Non-significant; A: extraction temperature (°C); B: pH; C: ethanol concentration (%); D: extraction time (min); TAC: total anthocyanin content (mg C3G/g D.W.); TPC: total phenolic content (mg GAE/g D.W.); AA: antioxidant activity (mMol Trolox/g D.W).

**Table 4:** Validation of the mathematical model.

Answer	Predicted Values	Confidence Interval 95%	Experimental Values
TAC, mg C3G/g D.W.	2.10	1.55–2.51	2.10 ± 0.02
TPC, mg GAE/g D.W.	20.30	18.65–22.26	20.60 ± 0.70
AA, mMol Trolox/g D.W.	19.05	17.72–20.23	19.02 ± 0.82

Total anthocyanin content (TAC), total phenolic content (TPC), and antioxidant activity (AA).

**Table 5:** Identification and quantitative characterization of the phenolic composition of purple carrot pulp powder by UHPLC–MS/MS.

No.	Compound Name	R.T. (min)	Formula	Exact Mass	Accurate Mass, [M-H]^−^	MS^2^ Fragments (*m*/*z*)	Concentration (mg/k, dw)	LD
Quantified phenolic compounds								
Phenolic acids/Hydroxybenzoic acids								
1.	Gallic acid	1.95	C_7_H_6_O_5_	170.0215	169.0133	169.0133; 125.0231	<0.50	0.50
2.	3,4-Dihydroxybenzoic acid	8.00	C_7_H_6_O_4_	154.0266	153.0183	109.0281	12.92 ± 1.21	0.83
3.	2,5-Dihydroxybenzoic acid	3.87	C_7_H_6_O_4_	154.0266	153.0183	109.0342; 153.0261	<0.64	0.63
4.	4-Hydroxybenzoic acid	6.74	C_7_H_6_O_3_	138.0316	137.0233	118.9650, 96.9588, 71.0124	4.75 ± 0.32	3.40
5.	Syringic acid	8.18	C_9_H_10_O_5_	198.0528	197.0446	182.0212, 166.9976, 153.0547, 138.0311, 123.0075	<1.35	1.40
Phenolic acids/Hydroxycinnamic acids								
6.	Caffeic acid	8.15	C_9_H_8_O_4_	180.0422	179.0341	135.0440	<0.81	0.80
7.	*p*-Coumaric acid	8.61	C_9_H_8_O_3_	164.0473	163.0392	119.0489	<1.10	1.10
8.	Synapic acid	8.92	C_11_H_12_O_12_	224.0684	223.0608	79.7560; 95.9510; 118.9651	<0.74	0.80
9.	Ferulic acid	8.85	C_10_H_10_O_4_	194.0579	193.0499	178.0262; 134.0361	<1.01	1.00
Tannins/Ellagitannins								
10.	Ellagic acid	9.72	C_14_H_6_O_8_	302.0062	300.9991	300.9991	12.92 ± 0.85	1.00
Flavonoids/Flavanols								
11.	Catechin	7.52	C_15_H_14_O_6_	290.0790	289.0718	109.0282, 125.0232, 137.0232, 151.0390	1.77 ± 0.10	0.80
12.	Epicatechin	8.13					<0.70	0.70
13.	Epigallocatechin-gallate, ECG	8.03	C_22_H_18_O_11_	458.0849	457.0776	169.0132, 125.0232, 289.0719	2.35 ± 0.21	0.17
Flavonoids/Flavanonols								
14.	Taxifolin	8.18	C_15_H_12_O_7_	304.0582	303.0511	147.0440; 257.0814	<0.79	0.79
Flavonoids/Flavanones								
15.	Naringin	9.06	C_27_H_32_O_14_	580.1791	579.1724	363,0721	3.20 ± 0.25	0.70
16.	Hesperidin	9.32	C_28_H_34_O_15_	610.1897	609.1826	377.0876	2.21 ± 2.21	0.80
17.	Pinocembrin	11.79	C_15_H_12_O_4_	256.0735	255.0661	213.0551; 151.0026; 107.0125	<0.85	0.90
Flavonoids/Flavones								
18.	Vitexin	8.97	C_21_H_20_O_10_	432.1056	431.0981	341.0664; 269.0454; 240.0422; 197.0606	<0.67	0.70
19.	Apigenin	11.97	C_15_H_10_O_5_	270.0528	269.0456	117.0333; 151.0027; 107.0126	<0.82	0.80
20.	Chrysin	13.73	C_15_H_10_O_4_	254.0579	253.0503	143.0491; 145.0284; 107.0125; 209.0603; 63.0226; 65.0019	<0.89	0.90
Flavonoids/Flavonols								
21.	Myricetin	8.08	C_15_H_10_O_8_	318.0375	317.0305	178.9986; 164.9263; 151.0036; 137.0244; 107.0125	1.38 ± 0.09	0.20
22.	Isorhamnetin	12.10	C_16_H_12_O_7_	316.0582	317.0665	300.0277	6.45 ± 0.77	0.60
23.	Rutin	9.40	C_27_H_30_O_16_	610.1533	609.1465	301.0352; 300.0276	2.26 ± 0.23	0.80
24.	Quercetin	10.72	C_15_H_10_O_7_	302.0426	301.0355	151.0226; 178.9977; 121.0282; 107.0125	12.92 ± 0.83	1.00
25.	Kaempferol		C_15_H_10_O_6_	286.0477	285.0403	151.0389; 117.0180	<0.98	1.00
26.	Galangin	9.23	C_15_H_10_O_5_	270.0528	269.0454	169.0650; 143.0491	<0.85	0.90
Flavonoids/Anthocyanins								
27.	Oenin	8.47	C_23_H_25_O_12_	493.1340	492.1239	492.1229; 329.0663	1.07 ± 0.94	0.60
Stilbenoid compounds								
28.	Isorhapontigenin	10.12	C_15_H_14_O_4_	258.0892	257.0819	241.0504; 125.0231; 175.0392; 217.0502; 175.0393	2.12 ± 0.13	1.20
29.	cis-Polydatin	8.77	C_20_H_22_O_8_	390.1314	389.1243	227.07088; 185.05998; 143.04909; 228.07426	3.44 ± 0.27	1.40
30.	trans-Polydatin	8.81					<1.40	1.40
Dihydrochalcones								
31.	Phlorizin	8.77	C_21_H_24_O_10_	436.1369	435.1296	107.0553	0.75 ± 0.03	0.70
32.	Phloretin	9.42	C_15_H_14_O_5_	274.0841	273.0768	93.0332; 121.0283	<0.28	0.30
Cyclohexanecarboxylic acid.								
33.	Shikimic acid	0.82	C_7_H_10_O_5_	174.0528	173.0447	162.8381; 160.8411; 164.8352	87.40 ± 8.04	3.14
Identified phenolic compounds								
Phenolic acids								
34.	Chlorogenic acid	7.82	C_16_H_18_O_9_	354.0950	353.0880	353.0879; 191.0552	identification	NQ
Phenolic acids derivatives								
35.	3-p-coumaroylquinic acid	9.15	C_16_H_18_O_8_	338.1001	337.0932	163.0390; 119.0495	identification	NQ
36.	3-O-feruloylquinic acid	8.51	C_17_H_20_O_9_	368.1107	367.1034	193.0499; 175.0387	identification	NQ
37.	5 Caffeoylquinic acid	6.79	C16H_18_O_9_	354.0950	353.0880	191.0549; 179.0338	identification	NQ
38.	Ferulic acid hexoside	7.95	C_16_H_20_O_9_	356.1107	355.1036	355.1036; 193.0495	identification	NQ
39.	Ferulic acid dihexoside	8.27	C_22_H_30_O_14_	518.1635	517.1562	517.1567; 355.1038; 341.1091; 193.0502;	identification	NQ
Flavonoids								
40.	Quercetin 3-O-galactoside	9.46	C_21_H_20_O_12_	464.0954	463.0881	179.0334; 193.0496;	identification	NQ
Acylated anthocyanins, Accurate mass, [M-H]+								
41.	cyanidin 3-xylosyl-galactoside	8.16	C_26_H_29_O_15_	580.1467	581.1503	287.0545; 177.0543; 135.0442	identification	NQ
42.	cyanidin3-xylosyl (sinapoylglucosyl)galactoside	8.20	C_43_H_49_O_24_	948.2534	949.2607	287.0547; 177.0543; 673, 1935	identification	NQ
43.	cyanidin3-xylosyl (coumaroylglucosyl)galactoside	8.44	C_41_H_45_O_22_	889.2402	890.2475	287.0547; 177.0543; 673, 1935; 889.2391	identification	NQ
44.	peonidin3-xylosyl (feruloylglucosyl)galactoside	8.48	C_43_H_49_O_23_	932.7826	933.2649	287.0547; 177.0543; 673, 1935; 889.2374	identification	NQ

Note: “<” indicates values below the method’s detection limit; NQ—not quantified.

**Table 6:** The value interval for the investigated factors and the encoded values.

Code	Independent Variables	Unit	Minimum Axial Level	−1	Center	+1	Maximum Axial Level
A	Temperature	°C	0.00	25.00	52.50	80.00	107.50
B	Time	min	0.00	20.00	40.00	60.00	80.00
C	Ethanol concentration	%	25.00	50.00	75.00	100.00	100.00 *
D	pH	-	0.00	1.00	2.50	4.00	5.50

* For ethanol concentration, the upper axial level was constrained to 100%.

## Data Availability

The original contributions presented in this study are included in the article. Further inquiries can be directed to the corresponding author.

## References

[1] Arscott S.A., Tanumihardjo S.A. (2010). Carrots of Many Colors Provide Basic Nutrition and Bioavailable Phytochemicals Acting as a Functional Food. Compr. Rev. Food Sci. Food Saf..

[2] Baranski R., Allender C., Klimek-Chodacka M. (2012). Towards Better Tasting and More Nutritious Carrots: Carotenoid and Sugar Content Variation in Carrot Genetic Resources. Food Res. Int..

[3] Sun T., Simon P.W., Tanumihardjo S.A. (2009). Antioxidant Phytochemicals and Antioxidant Capacity of Biofortified Carrots (*Daucus carota* L.) of Various Colors. J. Agric. Food Chem..

[4] Kammerer D., Carle R., Schieber A. (2003). Detection of Peonidin and Pelargonidin Glycosides in Black Carrots (*Daucus carota* ssp. *sativus* Var. *atrorubens* Alef.) by High-Performance Liquid Chromatography/Electrospray Ionization Mass Spectrometry. Rapid Commun. Mass Spectrom..

[5] Simon P.W. (2000). Domestication, Historical Development, and Modern Breeding of Carrot. Plant Breeding Reviews.

[6] Surles R.L., Weng N., Simon P.W., Tanumihardjo S.A. (2004). Carotenoid Profiles and Consumer Sensory Evaluation of Specialty Carrots (*Daucus carota*, L.) of Various Colors. J. Agric. Food Chem..

[7] Blando F., Calabriso N., Berland H., Maiorano G., Gerardi C., Carluccio M.A., Andersen Ø.M. (2018). Radical Scavenging and Anti-Inflammatory Activities of Representative Anthocyanin Groupings from Pigment-Rich Fruits and Vegetables. Int. J. Mol. Sci..

[8] Espinosa-Acosta G., Ramos-Jacques A.L., Molina G.A., Maya-Cornejo J., Esparza R., Hernandez-Martinez A.R., Sánchez-González I., Estevez M. (2018). Stability Analysis of Anthocyanins Using Alcoholic Extracts from Black Carrot (*Daucus carota* ssp. *Sativus* Var. *Atrorubens* Alef.). Molecules.

[9] Van Dokkum W., Frølich W., Saltmarsh M., Gee J. (2008). The Health Effects of Bioactive Plant Components in Food: Results and Opinions of the EU COST 926 Action. Nutr. Bull..

[10] Chhetri L., Rizwan M.D., Munkombwe S., Dorji N., Mutum E. (2022). Utilization and Characteristics of Black Carrot (*Daucus carrot* L.): Potential Health Benefits and Effect of Processing. Pharma Innov. Int. J..

[11] Rodríguez-Mena A., Ochoa-Martínez L.A., González-Herrera S.M., Rutiaga-Quiñones O.M., Morales-Castro J. (2021). Degradation Kinetics and Thermodynamic Analysis of Betalains on Microencapsulated Beetroot Juice Using Maltodextrin and Sweet Potato Starch. Sci. Agropecu..

[12] Pompeu D.R., Silva E.M., Rogez H. (2009). Optimisation of the Solvent Extraction of Phenolic Antioxidants from Fruits of *Euterpe oleracea* Using Response Surface Methodology. Bioresour. Technol..

[13] Amendola D., De Faveri D.M., Spigno G. (2010). Grape Marc Phenolics: Extraction Kinetics, Quality and Stability of Extracts. J. Food Eng..

[14] Sagar N.A., Pareek S., Sharma S., Yahia E.M., Lobo M.G. (2018). Fruit and Vegetable Waste: Bioactive Compounds, Their Extraction, and Possible Utilization. Compr. Rev. Food Sci. Food Saf..

[15] Hasbay I., Galanakis C.M., Galanakis C.M. (2018). Recovery Technologies and Encapsulation Techniques. Polyphenols: Properties, Recovery, and Applications.

[16] Xiang Y., Xiang M., Mao Y., Huang L., He Q., Dong Y. (2025). Insights into Structure-Antioxidant Activity Relationships of Polyphenol-Phospholipid Complexes: The Effect of Hydrogen Bonds Formed by Phenolic Hydroxyl Groups. Food Chem..

[17] Liao X., Greenspan P., Pegg R.B. (2021). Examining the Performance of Two Extraction Solvent Systems on Phenolic Constituents from U.S. Southeastern Blackberries. Molecules.

[18] Ma J., Chen C., Ma J., Ma W., Yang J. (2020). Analysis of Bioactive Compounds and Antioxidant Capacities in Different Varieties of Carrots. J. Phys. Conf. Ser..

[19] Leja M., Kamińska I., Kramer M., Maksylewicz-Kaul A., Kammerer D., Carle R., Baranski R. (2013). The Content of Phenolic Compounds and Radical Scavenging Activity Varies with Carrot Origin and Root Color. Plant Foods Hum. Nutr..

[20] Khazaei K.M., Jafari S.M., Ghorbani M., Kakhki A.H., Sarfarazi M. (2016). Optimization of Anthocyanin Extraction from Saffron Petals with Response Surface Methodology. Food Anal. Methods.

[21] Cacace J.E., Mazza G. (2003). Optimization of Extraction of Anthocyanins from Black Currants with Aqueous Ethanol. J. Food Sci..

[22] Backes E., Pereira C., Barros L., Prieto M.A., Genena A.K., Barreiro M.F., Ferreira I.C.F.R. (2018). Recovery of Bioactive Anthocyanin Pigments from *Ficus carica* L. Peel by Heat, Microwave, and Ultrasound Based Extraction Techniques. Food Res. Int..

[23] Pinelo M., Rubilar M., Jerez M., Sineiro J., Núñez M.J. (2005). Effect of Solvent, Temperature, and Solvent-to-Solid Ratio on the Total Phenolic Content and Antiradical Activity of Extracts from Different Components of Grape Pomace. J. Agric. Food Chem..

[24] Oleinits E., Hatem M.A., Deineka V., Chulkov A., Blinova I., Tretiakov M. (2019). Determination of Anthocyanins of Purple Carrot Two Cultivars.

[25] Pérez M.B., Carvajal S., Beretta V., Bannoud F., Fangio M.F., Berli F., Fontana A., Salomón M.V., Gonzalez R., Valerga L. (2023). Characterization of Purple Carrot Germplasm for Antioxidant Capacity and Root Concentration of Anthocyanins, Phenolics, and Carotenoids. Plants.

[26] Cacace J.E., Mazza G. (2003). Mass Transfer Process during Extraction of Phenolic Compounds from Milled Berries. J. Food Eng..

[27] Mantiniotou M., Athanasiadis V., Kalompatsios D., Lalas S.I. (2025). Optimization of Carotenoids and Other Antioxidant Compounds Extraction from Carrot Peels Using Response Surface Methodology. Biomass.

[28] Sabahi S., Abbasi A., Mortazavi S.A. (2024). Phenolic Components from Carrot (*Daucus carota* L.) Pomace: Optimizing the Extraction and Assessing Its Potential Antioxidant and Antimicrobial Activities. Heliyon.

[29] Yildiz E., Guldas M., Gurbuz O. (2020). Determination of In-Vitro Phenolics, Antioxidant Capacity and Bio-Accessibility of Kombucha Tea Produced from Black Carrot Varieties Grown in Turkey. Food Sci. Technol..

[30] Kosewski G., Górna I., Bolesławska I., Kowalówka M., Więckowska B., Główka A.K., Morawska A., Jakubowski K., Dobrzyńska M., Miszczuk P. (2018). Comparison of Antioxidative Properties of Raw Vegetables and Thermally Processed Ones Using the Conventional and Sous-Vide Methods. Food Chem..

[31] Stübler A.-S., Lesmes U., Juadjur A., Heinz V., Rauh C., Shpigelman A., Aganovic K. (2020). Impact of Pilot-Scale Processing (Thermal, PEF, HPP) on the Stability and Bioaccessibility of Polyphenols and Proteins in Mixed Protein- and Polyphenol-Rich Juice Systems. Innov. Food Sci. Emerg. Technol..

[32] Park H.Y., Hong J.W., Kim J.H., Kim Y.H., Kim J.W. (2023). Optimization of Ultrasound-Assisted Extraction Conditions for Extraction of Bioactive Compounds from Purple Carrot (*Daucus carota L.*) Using Response Surface Methodology. Food Sci. Technol..

[33] Yusuf E., Wojdyło A., Oszmiański J., Nowicka P. (2021). Nutritional, Phytochemical Characteristics and In Vitro Effect on α-Amylase, α-Glucosidase, Lipase, and Cholinesterase Activities of 12 Coloured Carrot Varieties. Foods.

[34] Yahia E.M. (2017). Fruit and Vegetable Phytochemicals: Chemistry and Human Health, 2 Volumes.

[35] Ahmad T., Cawood M., Iqbal Q., Ariño A., Batool A., Tariq R.M.S., Azam M., Akhtar S. (2019). Phytochemicals in *Daucus carota* and Their Health Benefits—Review Article. Foods.

[36] Kramer M., Maksylewicz-Kau A., Barański R., Nothnagel T., Carle R., Kammerer D.R. (2013). Effects of Cultivation Year and Growing Location on the Phenolic Profile of Differently Coloured Carrot Cultivars. J. Appl. Bot. Food Qual..

[37] El-Moslemany A.M., Abd-Elfatah M.H., Abd Elrahman W.M., Elgendy M.S.A., Ghamry H.I., El-Sherbiny M., Mojaddidi M.A., Alkeridis L.A., El-Wakeil N.H.M., Shukry M. (2025). Combatting BPA-Induced Neurotoxicity with Purple Carrot Extract (*Daucus carota*): Modulation of Key Neurotransmitters and Cellular Pathways in Albino Rats. J. Food Biochem..

[38] Gonçalves E.M., Pinheiro J., Abreu M., Brandão T.R.S., Silva C.L.M. (2010). Carrot (*Daucus carota L*.) Peroxidase Inactivation, Phenolic Content and Physical Changes Kinetics Due to Blanching. J. Food Eng..

[39] Pace B., Capotorto I., Cefola M., Minasi P., Montemurro N., Carbone V. (2020). Evaluation of Quality, Phenolic and Carotenoid Composition of Fresh-Cut Purple Polignano Carrots Stored in Modified Atmosphere. J. Food Compos. Anal..

[40] Ordóñez-Díaz J.L., Velasco-Ruiz I., Velasco-Tejero C., Pereira-Caro G., Moreno-Rojas J.M. (2024). Seasonal and Morphology Effects on Bioactive Compounds, Antioxidant Capacity, and Sugars Profile of Black Carrot (*Daucus carota* ssp. *sativus* Var. *atrorubens* Alef.). Foods.

[41] Lee J., Durst R.W., Wrolstad R.E. (2005). Determination of Total Monomeric Anthocyanin Pigment Content of Fruit Juices, Beverages, Natural Colorants, and Wines by the pH Differential Method: Collaborative Study. J. AOAC Int..

[42] Stoica F., Condurache N.N., Horincar G., Constantin O.E., Turturică M., Stănciuc N., Aprodu I., Croitoru C., Râpeanu G. (2022). Value-Added Crackers Enriched with Red Onion Skin Anthocyanins Entrapped in Different Combinations of Wall Materials. Antioxidants.

[43] Geana E.-I., Ciucure C.T., Tamaian R., Marinas I.C., Gaboreanu D.M., Stan M., Chitescu C.L. (2023). Antioxidant and Wound Healing Bioactive Potential of Extracts Obtained from Bark and Needles of Softwood Species. Antioxidants.

